# Clinical plausibility of eggshell membrane proteins for osteoarthritis: a study of solubility, digestibility, and immune response

**DOI:** 10.1007/s11010-026-05608-9

**Published:** 2026-07-01

**Authors:** Simona Gegel, Arun Kumar, Lilia Goudeva, Jürgen Borlak

**Affiliations:** 1 Centre for Pharmacology and Toxicology, Hannover Medical School, Carl-Neuberg-Str. 1, 30625 Hannover, Germany; 2 Institute of Transfusion Medicine and Transplant Engineering, Hannover Medical School, Carl-Neuberg-Str. 1, 30625 Hannover, Germany

**Keywords:** Osteoarthritis, Dietary supplements, Eggshell membrane proteins, Protein solubility and digestibility, Immunomodulation

## Abstract

Supplements are commonly used by osteoarthritis (OA) patients, and eggshell membrane (ESM) proteins are widely advertised as natural bioactive compounds for joint health. Here, we evaluated the solubility, digestibility, and immunomodulatory potential of commercial ESM supplements under physiologically relevant conditions to assess their plausibility as anti-inflammatory agents. We analyzed three commercial ESM formulations (NEM^®^, Torolis^®^, and Biova^®^) using standardized extraction protocols. Protein solubility was assessed in physiological saline and under simulated gastric (pH 2) and intestinal (pH 5.8) conditions with controlled temperature, agitation, and digestive enzyme exposure. We examined the role of disulfide-crosslinked proteins in insolubility using dithiothreitol (DTT) and evaluated digestibility of proteins by SDS–PAGE after treatment with pepsin, pancrelipase (Creon^®^), or sequential digestion. Released peptides were identified by MALDI-TOF mass spectrometry, and immunomodulatory effects were tested using human peripheral blood mononuclear cells (PBMC) by measuring cytokine release after stimulation with lipopolysaccharide or Phorbol 12-myristate 13-acetate/Ionomycin, in the presence or absence of Biova^®^. NEM^®^ and Torolis^®^ showed extremely low solubility (< 0.3%) and remained insoluble under all conditions, including after DTT treatment. Although Biova^®^ was highly soluble (~ 90%), simulated gastrointestinal digestion produced minimal peptide generation. Only non-physiological extraction yielded sporadic trace peptides, most lacking known anti-inflammatory relevance. In PBMC assays, Biova^®^ increased IL-6 after lipopolysaccharide stimulation, showed a non-significant TNFα reduction, and had no effect on IL-17 A or granzyme B. Together, commercial ESM supplements are largely insoluble, resistant to digestion, and show no meaningful anti-inflammatory activity in human immune cells. These findings challenge the plausibility of orally administered ESM as a therapeutic intervention for osteoarthritis.

**Supplementary Information:**

The online version contains supplementary material available at https://doi.org/10.1007/s11010-026-05608-9.

## Introduction

Osteoarthritis (OA), the most common form of arthritis, affects more than 500 million people worldwide [1]. It impairs musculoskeletal function through the degeneration of joint cartilage, causing pain, stiffness, swelling, and limited mobility. Recent American College of Rheumatology (ACR) guidelines prioritize non-pharmacological interventions (e.g., exercise, weight management) and approved medications like non-steroidal anti-inflammatory drugs (NSAIDs) for the treatment of OA. Apart from guideline driven therapeutic interventions, data indicate that 69% of OA-patients also take dietary supplements to better manage their condition [2]. Supplements include glucosamine and chondroitin, omega-3 fatty acids, curcumin, collagen among others. These supplements are thought to reduce pain and stiffness by providing building blocks for cartilage, to reduce inflammation, and function as antioxidants. However, evidence for supplements is mixed, and are not endorsed in OA-guidelines due to lack of sufficient evidence. In this regard, eggshell membrane (ESM) proteins have gained attention as a natural source of bioactive compounds that may help in the management of osteoarthritis and joint health. Although limited evidence from placebo-controlled randomized clinical trials [3–5] and meta-analysis [6, 7] indicates that ESM supplements may alleviate pain and stiffness and modestly improve musculoskeletal function in osteoarthritis (OA), these findings should be interpreted with caution. Specifically, the small cohort sizes, heterogeneous patient populations, and potential confounding from autoimmune conditions such as rheumatoid arthritis substantially limit the generalizability of existing findings. Furthermore, clinical trials have not yet established whether ESM supplementation can modulate circulating concentrations of key inflammatory mediators implicated in osteoarthritis pathogenesis. These mediators – including interleukin IL-17, IL-1β, IL-6 and the cytokine TNFα - play central roles in promoting cartilage degradation, synovial inflammation, and subchondral bone remodeling. In fact, the evidence on therapeutic efficacy is almost exclusively based on questionnaires such as the Western Ontario and McMaster Universities Osteoarthritis Index, i.e., a 24-item questionnaire used to measure pain, stiffness, and physical function. Given these limitations, the ACR OA-guidelines do not recommend ESM as a disease modifying therapy.

A critical question yet to be answered is the bioavailability of ESM derived proteins and their ability to repress inflammatory mediators in osteoarthritis. We therefore investigated the solubility and digestibility of different ESM formulations and subsequently tested one ESM formulation (Biova^®^) on cytokine release in peripheral blood mononuclear cells (PBMCs) of healthy volunteers following stimulation with lipopolysaccharide (LPS) and/or phorbol-12-myristate-13-acetate (PMA)/Ionomycin. We focused on IL-6, TNFalpha, IL-17 and Granzyme B which are known to be upregulated in OA-patients.

## Materials and methods

The ESM preparations NEM^®^ (Stratum Nutrition, Carthage, MO, USA, Lot #8100380), Torolis^®^ (TOROLIS^®^ S.L., Navarra, Spain, Lot #081019S) and Biova^®^ (Biova^®^, LLC, Johnston, IA, USA, Lot #Z8259-2) were obtained from Weber & Weber, Germany. These were dissolved in phosphate buffered saline (PBS) pH 7,4 or ddH_2_0 at a nominal concentration of 10 mg/ml. The samples were vigorously and repeatedly vortexed. For cell culture experiments, the ESM solutions were sterile filtered with a cellulose acetate membrane filter (Puradisc 30 syringe filter, 0.2 μm WHA10462200, Merck, Germany).

### ESM digest with pepsin and a cocktail of digestive enzymes

To mimic gastric digestion, ESM solutions were acidified with 5 M HCl to pH 2, followed by the addition of 1.3 mg/mL pepsin and incubation at 37 °C for 1–2 h. For intestinal conditions, samples were adjusted to pH 5.8 (NEM^®^, Torolis^®^, Biova^®^) or pH 8 (Biova^®^) and treated with 1 or 50 IU Creon^®^ (lipase, protease, amylase) at 37 °C, agitated at 250 rpm for 1–2 h.

### Dithiothreitol treatment of NEM^®^

Because of low solubility, NEM^®^ was mixed as a slurry (1 g in 7 mL PBS) and incubated with 1 mM DTT at room temperature for 24 h, then centrifuged for 10 min at 900 × g to collect the supernatant. Samples were adjusted to pH 2.0 for pepsin digestion, sonicated for 1 h, and protein concentrations determined by the Bradford assay. Poorly soluble NEM^®^ and Torolis^®^ samples were lyophilized and re-dissolved in SDS electrophoresis buffer. Control solutions of pepsin and Creon^®^ were prepared in PBS.

### SDS–PAGE analysis

Solutions of NEM^®^, Torolis^®^, and Biova^®^ were mixed with 4x SDS sample buffer (0.25 M Tris-HCl, pH 6.8, 0.05% bromophenol blue, 40% glycerol, 2% SDS). The mixtures were incubated at 95 °C for 5 min and loaded onto a 4% stacking gel over a 12–18% SDS–Tricine resolving gel containing 2% 2,2,2-trichloroethanol (TCE, T54801, Sigma-Aldrich, Germany).

We performed electrophoresis in a Bio-Rad chamber using Tris-Glycine buffer (25 mM Tris, 0.2 M glycine and 0.1% SDS) at 25 mA/gel. Following electrophoresis, the TCE-containing gels were illuminated under UV light for 2.5 min and visualized using a ChemiDoc XRS+ imaging system (Bio-Rad, Hercules, USA). Gels were also stained with Coomassie Brilliant Blue Dye (#1856209, Thermo Fisher Scientific, Waltham, USA) according to the manufacturer’s instructions.

### Protein identification by MALDI-TOF/MS

Protein identification was performed by MALDI-TOF/MS as previously described [8, 9]. Soluble proteins released from NEM^®^ and Biova^®^ were investigated based on a chemical extraction procedure described in [10]. Briefly, 1 g of eggshell membrane protein (ESM) was extracted in 10 mL of either 70% methanol containing 0.1% acetic acid or a buffer composed of 4 M guanidine hydrochloride (GdHCl), 20 mM EDTA, and 50 mM sodium acetate (pH 5.8). Extractions were performed by stirring with 10x volumes of the respective solutions at 4 °C for 24 h. Following centrifugation, the supernatants were collected, concentrated by lyophilization, and resuspended in a reduced volume. Protein concentrations were determined using the Bradford assay with bovine serum albumin (BSA) as the standard.

In addition, samples were digested with Creon^®^ for 60–120 min as described above. For MALDI-TOF/MS analysis, 1 µL of each sample at dilutions of 1:10, 1:50, or 1:100 was spotted onto an α-cyano-4-hydroxycinnamic acid (HCCA) matrix-coated target plate and overlaid with an ethanol/0.1% trifluoroacetic acid (TFA) solution (80:20, v/v). Peptide mass spectra were processed using BioTools software (Bruker Daltonics), and protein identification was performed using the MASCOT search engine (Matrix Science).

MS spectra were searched against the UniProt and NCBI protein databases with searches restricted to Gallus gallus. Proteins identified by at least two unique peptides and a minimum sequence coverage of ≥ 2% were retained for further analysis. In addition, proteins identified by a single unique peptide were included if the peptide provided ≥ 10% sequence coverage. All identified peptides were subsequently compared against the ESM protein database reported in [11] and assessed for their plausibility in contributing to anti-inflammatory activity and joint health.

### Isolation of peripheral blood mononuclear cells (PBMCs)

Two-milliliter aliquots of whole blood derived from routine blood donations by healthy volunteers at the Department of Transfusion Medicine, Hannover Medical School (see Supplementary Table S1A for donor information) were provided by Dr. Goudeva. Written informed consent was obtained from all donors, and the study was approved by the Ethics Committee of Hannover Medical School. All methods were performed in accordance with the relevant guidelines and regulations.

PBMCs were isolated by density gradient centrifugation. Briefly, 2 mL of whole blood was diluted with 4xPBS, pH 7.4 and carefully layered onto 3 mL of density gradient medium (Lymphoprep™, Cat# 07801/07811, Stemcell Technologies, Vancouver, Canada) in a 15 mL conical tube. The samples were centrifuged at 700 × g for 25 min at 18 °C with the centrifuge brake disengaged. Following centrifugation, the distinct interphase containing PBMCs were carefully collected and transferred to a new tube.

The PBMC suspension was brought to a final volume of 30 mL with PBS and centrifuged at 150 × g for 10 min at 4 °C without brake. The resulting cell pellet was resuspended in 5 mL of RPMI-1640 medium (Cat# 21875034, Gibco^TM^/Thermo Fisher Scientific, Waltham, USA) and washed by an additional centrifugation under the same conditions. The final pellet was resuspended in 5 mL of RPMI-1640 supplemented with 10% fetal bovine serum (FBS, Cat# 10500064, Gibco^TM^/Thermo Fisher Scientific). Prior to seeding, cell counts and viability were determined using a Bio-Rad automated cell counter (TC20, Bio-Rad).

In addition to freshly isolated PBMCs, cryopreserved human peripheral blood mononuclear cells were obtained from Lonza Bioscience, Germany (Cat# 4 W-270; see Supplementary Table S1B for donor details). These cryopreserved hPBMCs were used exclusively to investigate the immunomodulatory effects of the Biova^®^ alone treatment.

### Cell culture experiments

Freshly isolated PBMCs were seeded into 96-well plates at donor-dependent densities ranging from 18,000 to 62,000 cells per well and cultured in RPMI-1640 medium supplemented with 10% fetal bovine serum (FBS) for 24 h. Following the same protocol, the seeding density for the cryopreserved hPBMC ranged between 41,000 and 100,000 cells per well. Owing to the poor solubility of NEM^®^ and Torolis^®^, only Biova^®^ was included in subsequent experiments.

To induce cytokine release and evaluate the potential anti-inflammatory effects of Biova^®^, cells were stimulated for an additional 24 h with either lipopolysaccharide (LPS; 0.5 µg/mL; Cat# L2630, Sigma-Aldrich, Germany) or a combination of phorbol 12-myristate 13-acetate (PMA; 50 ng/mL; Cat# P1585, Sigma-Aldrich, Germany) and Ionomycin (2.5 µg/mL; Cat# sc-3592, Santa Cruz Biotechnology, Dallas, USA). In parallel, to assess possible intrinsic immunostimulatory properties, PBMCs were treated with Biova^®^ alone at a nominal concentration of 640 µg/mL. Dimethyl sulfoxide (DMSO; 0.01%) was used as the vehicle control. Following the 24 h stimulation period, culture supernatants were collected and stored at −80 °C until further analysis.

### Cytokine measurements

Cytokines were quantified using LEGENDplex™ multiplex immunoassays (#BLD-740267, BioLegend UK) on a BD FACSCanto™ cytometer. Values were normalized to cell numbers.

### Statistical analysis

Statistics were performed in GraphPad Prism 8. Data are mean ± SEM. Depending on distribution, comparisons included Kruskal–Wallis tests with multiple comparisons. Two-tailed p-values ≤ 0.05 (*),* ≤* 0.001 (**), and ≤ 0.0001 (****) were considered significant.

## Results

### Solubility of ESM proteins

**Fig. 1:**
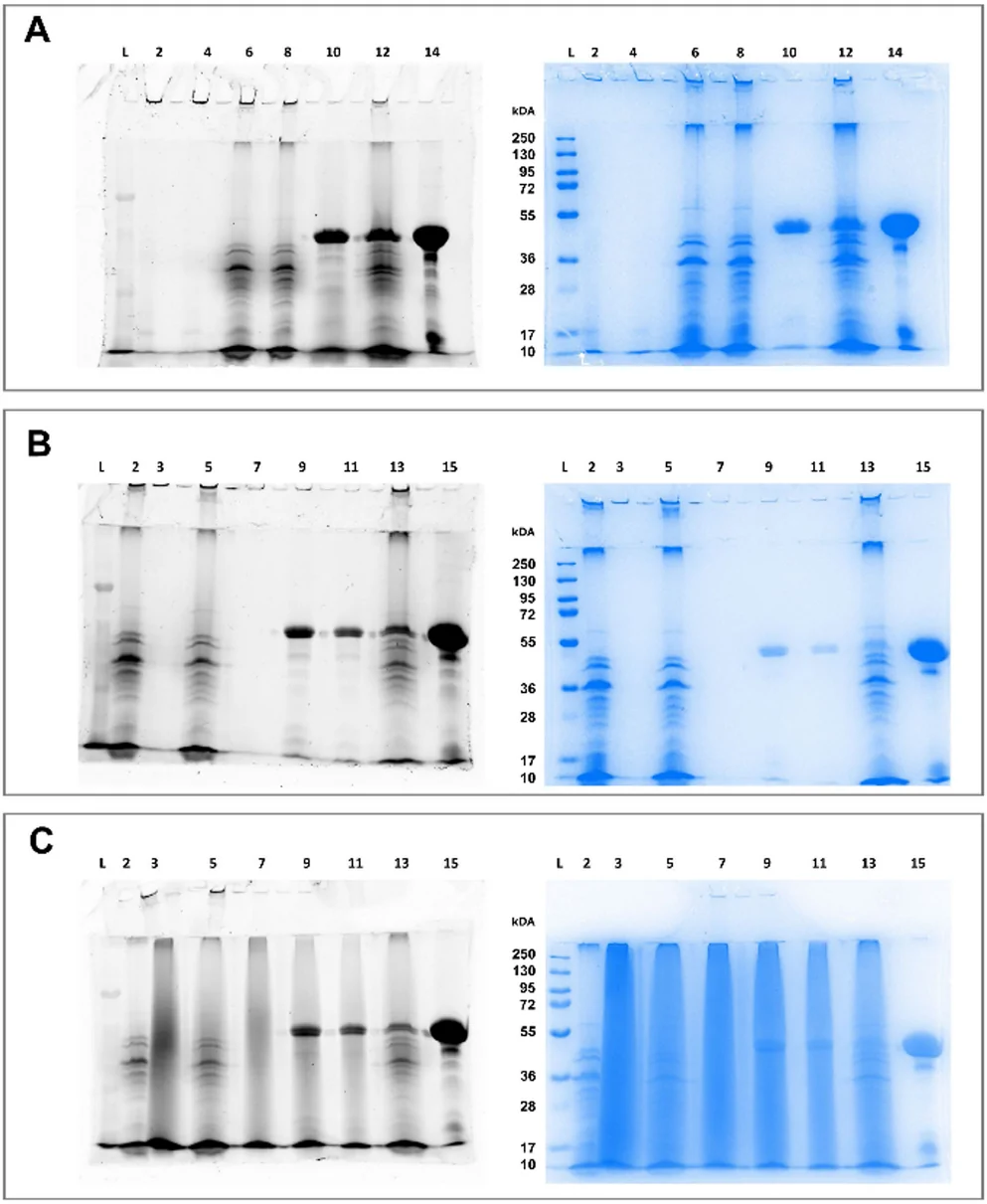
SDS-PAGE characterization of eggshell membrane protein solubility and digestibility. **A** Left panel: Representative 12% SDS–polyacrylamide gel containing TCE for total protein visualization. Lane L: molecular weight ladder. Lanes 2 and 4: NEM^®^ solution loaded at 15 µL and 7.5 µL, respectively, from stock at nominal 10 mg/mL. Lane 6: NEM^®^ following pancrelipase (Creon^®^) digestion at 37 °C for 1 h; all visible bands correspond to the Creon^®^ control. Lane 8: Creon^®^ control (50 IU). Lane 10: NEM^®^ (10 mg/mL) incubated with 1 mM DTT for 24 h at room temperature, acidified to pH 2, and digested with pepsin at 37 °C for 1 h; 1 mL of digest was lyophilized and reconstituted in SDS sample buffer. Bands observed are attributable to the pepsin control. Lane 12: NEM^®^ (10 mg/mL) incubated with 1 mM DTT for 24 h followed by sequential digestion with pepsin (pH 2) and Creon^®^ (pH 5.8) at 37 °C for 1 h each; 1 mL of digest was lyophilized and reconstituted in SDS sample buffer. Lane 14: Pepsin control (75 µg). Right panel: The same gel was subsequently stained with Coomassie Brilliant Blue to confirm protein band patterns observed by TCE fluorescence. **B** Left panel: Representative 12% SDS–polyacrylamide gel containing TCE for total protein visualization. Lane L: Molecular weight ladder. Lane 2: Creon^®^ control (50 IU) Lane 3: Torolis^®^ sample; 7.5 µL of a nominal 10 mg/mL stock solution loaded onto the stacking gel. Lane 5: Torolis^®^ after pancrelipase (Creon^®^) digestion at 37 °C for 1 h. All visible bands correspond to the Creon^®^ control. Lane 7: Torolis^®^ (10 mg/mL) ultrasonicated for 1 h, centrifuged, and 15 µL of the supernatant loaded. No protein bands detected. Lane 9: Torolis^®^ (10 mg/mL) acidified to pH 2 and digested with pepsin at 37 °C for 1 h; 15 µL loaded. All observed bands originate from the pepsin control. Lane 11: Torolis^®^ (10 mg/mL) treated as in Lane 9, but with 7.5 µL loaded. All observed bands originate from the pepsin control. Lane 13: Torolis^®^ (10 mg/mL) acidified to pH 2 and digested with pepsin at 37 °C for 1 h, followed by pancrelipase (Creon^®^, pH 5.8) digestion at 37 °C for 1 h. Lane 15: Pepsin control (75 µg). Right panel: The same gel was subsequently stained with Coomassie Brilliant Blue to confirm protein band patterns observed by TCE fluorescence. **C** Left panel: Representative 12% SDS–polyacrylamide gel containing TCE for total protein visualization. Lane L: Molecular weight ladder. Lane 2: Creon^®^ control (50 IU) Lane 3: Biova^®^ sample; 160 µg protein loaded onto the stacking gel. Lane 5: Biova^®^ after pancrelipase (Creon^®^) digestion at 37 °C for 1 h. All visible bands correspond to the Creon^®^ control. Lane 7: Biova^®^ (10 mg/mL) ultrasonicated for 1 h, centrifuged, and 160 µg protein loaded onto the stacking gel. No protein bands detected. Lane 9: Biova^®^ (10 mg/mL) acidified to pH 2 and digested with pepsin at 37 °C for 1 h; 160 µg protein loaded. All observed bands originate from the pepsin control. Lane 11: Biova^®^ (10 mg/mL) treated as in Lane 9, but half of the protein (80 µg) loaded onto the stacking gel. All observed bands originate from the pepsin control. Lane 13: Biova^®^ (10 mg/mL) acidified to pH 2 and digested with pepsin at 37 °C for 1 h, followed by pancrelipase (Creon^®^; pH 5.8) digestion at 37 °C for 1 h. 80 µg protein loaded onto the stacking gel. Lane 15: Pepsin control (75 µg). Right panel: The same gel was subsequently stained with Coomassie Brilliant Blue to confirm protein band patterns observed by TCE fluorescence

Slurries of the different formulations were prepared to assess protein solubility. Both NEM^®^ and Torolis^®^ exhibited extremely low protein solubilities of 0.18 ± 0.03% and 0.26 ± 0.03%, respectively, indicating that more than 99.7% of their total protein content remained insoluble under physiological saline conditions. This pronounced insolubility was further supported by SDS-PAGE analysis (Fig. 1), which revealed only faint and sparse protein bands in the molecular weight range of 15–45 kDa. In contrast, Biova^®^ exhibited a solubility of 90 ± 9.42%, demonstrating excellent solubility.

We evaluated the solubility of ESM under physiological conditions by adjusting the pH to mimic those found in the stomach (pH 2) and intestine (pH 5.8 or pH 8) during normal protein digestion. Temperature and digestive enzymes were carefully controlled, and samples were continuously agitated to simulate the dynamic processes of gastric emptying and peristalsis.

Because ESM proteins are highly cross-linked and rich in cysteine residues [12], we additionally treated NEM^®^ with dithiothreitol (DTT) to disrupt disulfide bonds and enhance solubility. Nevertheless, even after 24 h of DTT treatment, the protein solubility remained unchanged as evidenced by the Bradford assay.

### SDS-PAGE of ESM proteins

To evaluate the size distribution of soluble proteins, SDS–PAGE analyses were performed using 12% and 15% polyacrylamide gels. Increasing the acrylamide concentration decreases gel pore size, thereby improving the resolution of low-molecular-weight proteins. The main results are presented for the 12% gels (Fig. 1), while representative data obtained with the 15% gels are provided in supplementary Figure S1. All samples were analyzed under identical experimental conditions on both gel types. Because of its high solubility, Biova^®^ was additionally analyzed using an 18% gel to further improve the separation of low-molecular-weight proteins; representative results are shown in supplementary Figure S2. As shown in Fig. 1A, lane 2, the NEM^®^ solution exhibited approximately five faint bands between 15 and 45 kDa. After a two-fold dilution (lane 4), only a single ~ 15 kDa band remained detectable. Lane 6 displays the protein profile of the NEM^®^ solution following incubation with the Creon^®^ digestive enzyme mix. Comparison with the Creon^®^ control (lane 8) indicates that all visible bands are attributable to Creon^®^. Taken together, these data suggest that, in addition to its poor solubility in physiological saline, NEM^®^ is essentially resistant to digestion under physiological conditions.

Comparable findings were obtained when NEM^®^ was treated with DTT for 24 h and subsequently incubated with pepsin after pH adjustment. To increase analytical sensitivity, the NEM^®^ solution was lyophilized after DTT and pepsin treatment (detection limit 0.00005 mg/protein/lane). As shown in lane 10, the only detectable bands correspond to pepsin, consistent with the pepsin control in lane 14. Finally, lane 12 presents the same digest supplemented with 50 IU Creon^®^; once again, all visible protein bands derive from the added digestive enzymes rather than from NEM^®^.

We applied the same procedure to Torolis^®^ (Fig. 1B), additionally subjecting the samples to sonication to enhance solubility. As shown in lanes 3 and 7, no protein bands were detected, indicating that Torolis^®^ is insoluble in physiological saline. Likewise, treatment of Torolis^®^ with 50 IU Creon^®^ did not generate any new protein bands (lane 5); the bands that are present correspond solely to Creon^®^, as confirmed by the Creon^®^ control in lane 2. Pepsin digests of Torolis^®^ yielded a similar outcome: the bands observed in lanes 9 and 11 match those of the pepsin control (lane 15), demonstrating that they originate from pepsin rather than from Torolis^®^. Taken together, and consistent with our findings for NEM^®^, Torolis^®^ appears indigestible under physiological conditions.

Figure 1C shows a representative SDS–PAGE image of Biova^®^. Although Biova^®^ is highly soluble, it appeared as a broad smear without distinct protein bands (lane 3). Following incubation with Creon^®^, the bands observed in lane 5 correspond exclusively to Creon^®^ proteins, as confirmed by the Creon^®^ control in lane 2. Pepsin treatment produced a similar outcome: the bands detected in lanes 9 and 11 matched those of the pepsin control (lane 15), indicating that they represent pepsin-derived proteins rather than Biova^®^.

However, analysis using 18% acrylamide gels (supplementary Figure S2) revealed the presence of a small number of low-molecular weight bands following pepsin digestion (highlighted by the green box), which subsequently disappeared after additional Creon^®^ treatment (highlighted by the red box).

Finally, the protein bands present in lane 13 of Fig. 1C arise from the combined addition of pepsin and Creon^®^, not from Biova^®^.

Taken together, these findings demonstrate that all tested ESM preparations are indigestible under physiological conditions.

### Identification of proteins by MALDI-TOF/MS

Peptides derived from eggshell membrane (ESM) proteins were detected only after chemical extraction followed by enzymatic hydrolysis with trypsin and Creon^®^, i.e., conditions that do not reflect physiological digestion. Under these non-physiological conditions, MALDI-TOF/MS analysis identified 14 peptides in the NEM^®^ preparation and 28 peptides in the Biova^®^ preparation. (Table 1 and Supplementary Tables S2 and S3).

Despite the harsh extraction conditions, only trace amounts of peptides were recovered from ESM, reinforcing the notion that ESM proteins exhibit extremely poor solubility, particularly under physiological conditions.

Notwithstanding and based on chemical extraction (but not under physiological conditions), unique peptides corresponding to osteocalcin, lysozyme C, and platelet-activating factor acetylhydrolase were identified in Biova^®^ preparations. These proteins are plausible biomolecules with anti-inflammatory and joint health–modifying potential. In addition, peptides related to the adenosine A2B receptor were detected. This receptor is expressed on intestinal epithelial cells, whereas myosin light chain 1 may influence intestinal barrier permeability. Peptides marked with an asterisk have been independently reported as ESM proteins (Table 1).

**Table 1:** MALDI-TOF/MS identification of proteins from BIOVA following chemical extraction and enzymatic digestion

S.No	UniProt ID	Protein	Protein coverage (%)	Unique peptide sequence identified	Biological role	Citation
1	Q540H7	Adenosine receptor A2b	1	MNTMK	Adenosine A2B receptor suppresses NF-κB, TNF-α, IL-6	PMID: 9,244,356
2	Q9PRL8	Acyl-CoA-binding protein	2	AAEEVKELK	Lipid metabolism and signaling	
3	P21611	Beta-2-microglobulin	19	DGVPMEGAQYSDMSFNDDWTFQR	Expression reflects immune cell activation	PMID: 1,538,136
4	Q5ZJA9	BLOC-1-related complex subunit 5	18	IQMGIDQTVPLMERLNSMLPESEQLEPFSMKPDR	Lysosomal positioning; cartilage and chondrocyte metabolism	PMID: 15,642,098
6	Q5ZHP3	Cilia- and flagella-associated protein	4	LPFLVMIIK	Ciliary function; cilia involved in cartilage mechanosensing	
5	Q13901	Nuclear nucleic acid-binding protein C1D	3	TYMNK	DNA damage and stress response linked to inflammation	PMID: 10,362,552
7	A0AVX7	Calcineurin B homologous protein	31	KAPAGLAEEINFEDFLTIMSYFRPIEMDMDEER; APAGLAEEINFEDFLTIMSYFRPIEMDMDEER	Influences cell–matrix interactions	PMID: 19,345,287
8	Q90964	Forkhead box protein G1	2	AEALAGK; AKLAFK	Transcription factor	
9	O93512	GDNF family receptor alpha	2	CLDAAK; SKCHK	Neurotrophic signaling; pain and joint innervation	PMID: 9,740,802
10	Q6QLR1	Gallinacin-9*	47	MRILFFLVAVLFFLFQAAPAYSQEDADTLACR	Emphasizing immune–ECM crosstalk	PMID: 28,270,742
11	P84247	Histone H3.3	27	MARTK; FQSAAIGALQEASEAYLVGLFEDTNLCAIHAK	Epigenetic regulation of inflammatory gene expression	PMID: 3,627,987
12	P01994	Hemoglobin subunit alpha	16	IAGHAEEYGAETLERMFTTYPPTK	Oxygen delivery; hypoxia influences cartilage degeneration	PMID: 1,656,392
13	Q5ZJ26	protein KIAA1671	1	CDFSK	Involved in cell signaling or cytoskeletal interactions	
14	P00698	Lysozyme C*	15	NTDGSTDYGILQINSRWWCNDGR	Innate immune regulation, antimicrobial defense reduces inflammatory burden	PMID: 25,994,146
15	P02606	Myosin light chain 1	14	HVLR; LMAGQEDANGCINYEAFVKHIMAN		PMID: 6,772,448
16	Q2YHT5	CD200 receptor 1	15	NTAWTIVLLTSAAVMGASGISRVSANLGHSTVMTCPNR	Cell-surface receptor linked to immune–tissue interaction	PMID: 18,062,907
17	D0PRN2	Neurexin-1-beta	2	IGGKER; SSNKNK	Cell adhesion and tissue organization	PMID: 19,926,856
18	P15771	Nucleolin OS	2	MAPPPK; QGTEVDGR	Intranuclear packaging of preribosomal	PMID: 2,914,325
19	Q5ZJR3	Oligosaccharyltransferase complex	2	VFMR	ER stress and protein folding influence inflammatory signaling	PMID: 8,175,777
20	P02822	Osteocalcin	9	SAKAFISHR	Bone formation, mineralization; key marker of bone and joint health	PMID: 6,792,200
21	Q90678	Platelet-activating factor acetylhydrolase*	7	VFMK; IPAGKGPHAVGCTDLMTGDAAEGSFLR	Degrades platelet-activating factor	PMID: 7,592,717
22	Q9PWE0	Pituitary homeobox	1	MSCMK		
23	Q6ITC7	Small ribosomal subunit	3	MHAPGK		
24	Q9DG09	Steroidogenic acute regulatory protein	3	NVTGLR; QRMAR	Steroidogenesis affects inflammatory and joint physiology	PMID: 11,064,158
25	Q90627	Trypsin I-P1	2	TTMSSN	Proteolytic cleavage	PMID: 7,733,885
26	Q90628	Trypsin I-P38	2	TTMSSN	Proteolytic cleavage	PMID: 7,733,885
27	Q5ZM35	Twinfilin-2	4	MLYAATR; GPGENGEDS	Actin remodeling in fibroblasts and synoviocytes	PMID: 19,955,359
28	Q5ZL74	Vesicle-associated membrane protein	2	AMCMK	Vesicle trafficking in immune and inflammatory cells	PMID: 19,138,172

* independently identified ESM proteins with appropriate citations

Shown are the unique sequences of soluble peptides released from Biova following chemical extraction, lyophilization-based concentration, and enzymatic digestion

Chemical extraction of NEM^®^ preparations revealed unique peptides corresponding to glutamine synthase (GLNA) and small ubiquitin-like modifier 3 (SUMO3), both of which are key effectors in immune responses (Table 2). Additional unique peptides were assigned to Rho family-interacting cell polarization regulator 2, inhibitor of growth, apoptosis inhibitor 5, CYFIP-related Rac1 interactor A, and phosphatidylinositol-4-phosphate 5-kinase type 1 beta, all of which are known to modulate immune response. However, none of these proteins are established constituents of ESM. Importantly, most of the proteins identified are unlikely to be absorbed intact from the human gastrointestinal tract. Typically, dietary proteins are degraded into small peptides and amino acids prior to crossing the intestinal barrier.

**Table 2:** MALDI-TOF/MS identification of proteins from NEM following chemical extraction and enzymatic digestion

S.No	UniProt ID	Protein	Protein coverage (%)	Unique peptide sequence identified	Biological role	Citation
1	Q5ZMW3	Apoptosis inhibitor 5	2	NVHSTK; ASAGPKR	Anti-apoptotic; supports survival in inflammatory environments	PMID: 17,112,319
2	Q5ZI04	CYFIP-related Rac1 interactor A	3	MGNLLK; EQALAK	Actin-Rac1 signaling; tissue organization	
3	P16850	Glutamine synthetase	2	FILHR; NVGHEK	Chondrocyte metabolism and cartilage homeostasis	PMID: 19,895,308
4	Q5ZKY4	Inhibitor of growth	2	FTEMR; ASYEAFK	Epigenetic control of tissue differentiation	PMID: 17,692,502
5	Q5ZKY8	Mitochondrial ribosomal protein L15	3	KPERR; GQPIPK	Mitochondrial support for tissue metabolism	PMID: 31,888,468
6	P18444	N-myc	2	NEKAAK; IEYKR	Proliferation-associated inflammation	PMID: 2,183,017
7	Q90623	Myosin phosphatase-targeting subunit 1	1	SGSSYAR; RPREK	Regulates myosin phosphatase; smooth muscle tone, joint vasculature	PMID: 7,982,954
8	Q5ZIN1	Nuclear distribution protein C	3	LAKEAK; LRVGLK	Microtubule function; cell division and tissue maintenance	PMID: 21,771,589
9	P48440	Dolichyl-diphosphooligosaccharide	3	APTIVGKKALN; QKAAPGSKRY	ER protein quality control in matrix-producing cells	
10	P48440	Dolichyl-diphosphooligosaccharide	2	APTIVGK; AAPGSKR	ER protein quality control in matrix-producing cells	
11	Q5ZJ58	Phosphatidylinositol-4-phosphate 5-kinase type 1 beta	1	SHKGEK; LMKK	Signal transduction in stromal cells	PMID: 20,622,009
12	Q5F3L9	RHO family-interacting cell polarization regulator 2	3	GLAGFAR; IEVNGK; VTPRAR	Cytoskeletal dynamics in fibroblasts and synoviocytes	
13	P62985	Ubiquitin-60 S ribosomal protein	7	YNCDK	Protein turnover; stress-responsive	PMID: 2,850,543
14	Q5ZHQ1	SUMO3	42	QGLSMR; FDGQPINEAD; TPAQLEMEDED; TIDVFQQQTGGLC	SUMOylation suppresses NF-κB, AP-1, and inflammatory transcription	

Shown are the unique sequences of soluble peptides released from NEM following chemical extraction, lyophilization-based concentration, and enzymatic digestion

### Impact of Biova^®^ on LPS- and PMA/Ionomycin-induced cytokine production in PBMCs

**Fig. 2:**
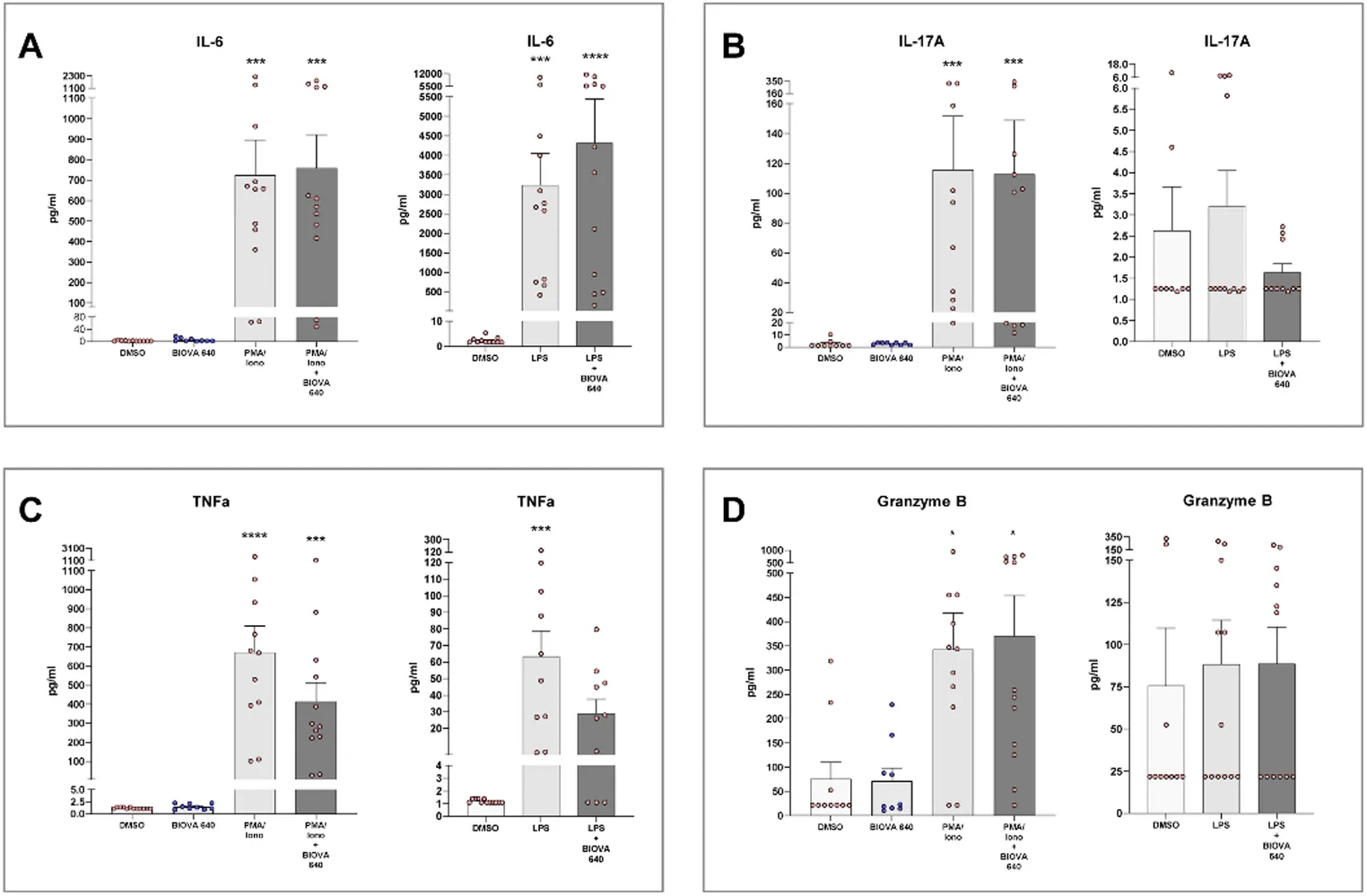
Effects of Biova^®^ on cytokines and Granzyme B release from peripheral blood mononuclear cells (PBMCs) of healthy donors. PBMCs were stimulated for 24 h with Biova^®^ alone, PMA/Ionomycin, or LPS, with or without Biova^®^ co-treatment. Shown are the concentrations of IL-6, IL-17 A, TNFα, and granzyme B in the cell culture supernatants. Biova^®^ alone treatment of PBMCs had no immunomodulatory effect. **A** Both stimuli induced robust IL-6 release, and Biova^®^ co-treatment increased IL-6 levels following LPS stimulation, but this change was not statistically significant. **B** PMA/Ionomycin, but not LPS, significantly elevated IL-17 A release. Biova^®^ co-treatment did not alter IL-17 A production. **C** Both stimuli significantly increased TNFα secretion. Co-treatment with Biova^®^ resulted in a non-significant reduction in TNFα levels. **D** PMA/Ionomycin, but not LPS, significantly enhanced Granzyme B release. Biova^®^ co-treatment had no detectable effect on Granzyme B secretion

Figure 2 summarizes the results for four selected cytokines. First, we evaluated whether Biova^®^ treatment alone influenced cytokine release; however, no intrinsic immunostimulatory effect was observed. In contrast, LPS elicited a robust induction of IL-6 and a modest increase in TNFα but did not stimulate the release of IL-17 A or Granzyme B. Co-treatment with Biova^®^ did not significantly reduce cytokine production. Instead, IL-6 levels increased by approximately 35%, whereas TNFα levels were reduced by about 50% relative to LPS alone. However, none of these changes reached statistical significance. It should be noted that TNFα is only weakly induced by LPS - approximately 10% of the level observed following PMA/Ionomycin stimulation. Because LPS does not induce Granzyme B, the minor fluctuations observed for this cytokine are likely incidental.

PMA/Ionomycin treatment significantly increased TNFα, and co-treatment with Biova^®^ produced a non-significant ~ 40% decrease. PMA/Ionomycin also significantly elevated IL-6, IL-17 A, and Granzyme B, yet Biova^®^ had no measurable effect on any of these responses.

Overall, although Biova^®^ co-treatment tended to reduce TNFα under both stimulation conditions, these effects were not statistically significant. Moreover, LPS-induced IL-6 release was higher in Biova^®^ co-treated PBMCs. Collectively, these findings suggest that ESM/Biova^®^ is unlikely to mitigate OA-related cytokine responses in patients.

## Discussion

We evaluated the solubility, digestibility, and immunomodulatory potential of commercially available eggshell membrane (ESM) supplements. For two of the three formulations, we observed extremely poor solubility under physiologically relevant conditions, and for all formulations a striking resistance to gastrointestinal digestion, and no significant anti-inflammatory effects in primary human immune cells. These findings call into question the biological plausibility of oral ESM as a therapeutic or disease-modifying agent in osteoarthritis (OA).

NEM^®^ and Torolis^®^ were almost entirely insoluble in physiological saline, with < 0.3% of total protein entering solution. Even when digestion was simulated under controlled gastric and intestinal conditions - including pH, temperature, enzymatic exposure, and mechanical agitation - solubility did not increase. Disruption of disulfide bonds via prolonged DTT treatment, included to mimic reductive processes, sometimes attributed to gut microbial activity, also failed to liberate additional protein. Biova^®^ showed high solubility, yet lacked discrete proteins or peptides upon SDS–PAGE analysis, suggesting the soluble fraction consists primarily of amorphous, non-bioavailable aggregates.

Consistent with this, none of the ESM formulations produced appreciable amounts of detectable digestion products following exposure to pepsin, pancrelipase, or both. Basically all visible bands in PAGE analysis (Fig. 1) originated from the digestive enzymes themselves. These findings reflect the highly cross-linked, cysteine-rich architecture of ESM proteins which form a keratin-like matrix inherently resistant to proteolysis [12].

Because systemic bioavailability appeared unlikely, we tested whether Biova^®^ could exert direct immunomodulatory activity. However, Biova^®^ treatment alone had no immunomodulatory effect, and co-treatment with Biova^®^ failed to suppress cytokine release from human PBMCs across two robust stimulation models. Reductions in TNFα did not reach statistical significance, and IL-6 was increased under LPS stimulation. Overall, these results do not support a direct anti-inflammatory effect that would be relevant in osteoarthritis.

We also compared proteins identified in the present study with those reported in a comprehensive ESM proteome mapping analysis that employed a range of extraction and solubilization conditions [11]. As noted by the authors, more than half of the reported proteins were detectable only under harsh extraction conditions. However, such conditions are physiologically irrelevant. Under normal physiological circumstances, ESM proteins exhibit extremely low solubility and limited digestibility. Consequently, proteins identified exclusively following chemical extraction are unlikely to exert therapeutic effects in vivo, and intact proteins do not cross the intestinal barrier. Instead, proteins are subjected to extensive gastrointestinal digestion.

Ultimately, the clinical evidence supporting the proposed anti-inflammatory effects of ESM remains sparse and is confounded by several methodological limitations, such as small cohort sizes, brief intervention periods, and a heavy reliance on subjective outcome measures. Although some trials report improvements in pain and joint function, claims of anti-inflammatory activity are largely predicated on patient-reported outcomes rather than objective biological endpoints. Note most clinical investigations have failed to incorporate direct assessments of key inflammatory mediators (e.g., TNFα, IL-1ß, and IL-6) or other validated biomarkers required to substantiate a mechanistic effect. Consequently, despite documented symptomatic benefits, robust experimental or clinical data demonstrating that ESM exerts clinically meaningful anti-inflammatory effects - or elucidating its precise mechanism of action in osteoarthritis - are currently lacking. Assertions regarding its therapeutic efficacy in mitigating inflammation must therefore be considered preliminary and insufficiently supported by the available literature.

### Study limitations

Several limitations warrant consideration. First, our digestion models, while reflective of major physiological parameters, cannot fully recapitulate the complexity of the human gastrointestinal tract. Second, only protein-based mechanisms were examined; we did not evaluate potential effects of minor non-protein constituents present in ESM formulations. Third, although PBMC assays are widely used to assess innate and adaptive immune activation, they may not capture all cell types or joint-specific microenvironmental factors relevant to OA pathophysiology. Finally, the gut microbiome represents an additional potential mediator of bioavailability. Nonetheless, the rigid disulfide-rich crosslinked ESM proteins are typically resistant to cleavage by human commensals.

## Conclusions

Our findings suggest that orally administered ESM proteins are mostly insoluble, indigestible, and lack direct immunomodulatory activity, limiting their biological plausibility as anti-inflammatory or disease-modifying agents in OA.

## Supplementary Information

Below is the link to the electronic supplementary material.


Supplementary Material 1



Supplementary Material 2



Supplementary Material 3



Supplementary Material 4


## Data Availability

All data generated or analyzed during this study are included in this published article and its supplementary information files.

## Abbreviations

ESM: Eggshell membrane
DDT: Dithiothreitol
LPS: Lipopolysaccharide
NSAIDs: Non-steroidal anti-inflammatory drugs
PMA: Phorbol 12-myristate 13-acetate
OA: Osteoarthritis
PBMC: Peripheral blood mononuclear cells
