# Supplementary material for: Clinical plausibility of eggshell membrane proteins for osteoarthritis: a study of solubility, digestibility, and immune response
Source: Mol Cell Biochem. 2026 Jul 1;481(8):3497–507. doi: 10.1007/s11010-026-05608-9 (PMC13525073; doi:10.1007/s11010-026-05608-9)
Supplement: Supplementary file 1 [file 11010_2026_5608_MOESM1_ESM.docx]

**SUPPLEMENTARY INFORMATION**

**Supplementary Figure S1: SDS-PAGE characterization of eggshell membrane protein solubility and digestibility.**

**(A) Left panel:** Representative 15% SDS–polyacrylamide gel containing TCE for total protein visualization. **L**: molecular weight ladder. **Lanes 2 and 4**: NEM® solution loaded at 15 µL and 7.5 µL, respectively, from stock at nominal 10 mg/mL. **Lane 6**: NEM® following pancrelipase (Creon®) digestion at 37°C for 1 h; all visible bands correspond to the Creon® control. **Lane 8**: Creon® control (50 IU). **Lane 10**: NEM® (10 mg/mL) incubated with 1 mM DTT for 24 h at room temperature, acidified to pH 2, and digested with pepsin at 37°C for 1 h; 1 mL of digest was lyophilized and reconstituted in SDS sample buffer. Bands observed are attributable to the pepsin control. **Lane 12**: NEM® (10 mg/mL) incubated with 1 mM DTT for 24 h followed by sequential digestion with pepsin (pH 2) and Creon® (pH 5.8) at 37°C for 1 h each; 1 mL of digest was lyophilized and reconstituted in SDS sample buffer. **Lane 14**: Pepsin control (75 µg).

**Right panel:** The same gel was subsequently stained with Coomassie Brilliant Blue to confirm protein band patterns observed by TCE fluorescence.

**(B) Left panel:** Representative 15% SDS–polyacrylamide gel containing TCE for total protein visualization. **L:** Molecular weight ladder. **Lane 2:** Creon® control (50 IU) **Lane 3:** Torolis® sample; 7.5 µL of a nominal 10 mg/mL stock solution loaded onto the stacking gel. **Lane 5:** Torolis® after pancrelipase (Creon®) digestion at 37°C for 1 h. All visible bands correspond to the Creon® control. **Lane 7:** Torolis® (10 mg/mL) ultrasonicated for 1 h, centrifuged, and 15 µL of the supernatant loaded. No protein bands detected. **Lane 9:** Torolis® (10 mg/mL) acidified to pH 2 and digested with pepsin at 37°C for 1 h; 15 µL loaded. All observed bands originate from the pepsin control. **Lane 11:** Torolis® (10 mg/mL) treated as in Lane 9, but with 7.5 µL loaded. All observed bands originate from the pepsin control. **Lane 13:** Torolis® (10 mg/mL) acidified to pH 2 and digested with pepsin at 37°C for 1 h, followed by pancrelipase (Creon®, pH 5.8) digestion at 37°C for 1 h. **Lane 15:** Pepsin control (75 µg).

**Right panel:** The same gel was subsequently stained with Coomassie Brilliant Blue to confirm protein band patterns observed by TCE fluorescence.

**(C) Left panel:** Representative 15% SDS–polyacrylamide gel containing TCE for total protein visualization. **L:** Molecular weight ladder. **Lane 2:** Creon® control (50 IU) **Lane 3:** Biova® sample; 160 µg protein loaded onto the stacking gel. **Lane 5:** Biova® after pancrelipase (Creon®) digestion at 37°C for 1 h. All visible bands correspond to the Creon® control. **Lane 7:** Biova® (10 mg/mL) ultrasonicated for 1 h, centrifuged, and 160 µg protein loaded onto the stacking gel. No protein bands detected. **Lane 9:** Biova® (10 mg/mL) acidified to pH 2 and digested with pepsin at 37°C for 1 h; 160 µg protein loaded. All observed bands originate from the pepsin control. **Lane 11:** Biova® (10 mg/mL) treated as in Lane 9, but half of the protein (80 µg) loaded onto the stacking gel. All observed bands originate from the pepsin control. **Lane 13:** Biova® (10 mg/mL) acidified to pH 2 and digested with pepsin at 37°C for 1 h, followed by pancrelipase (Creon®; pH 5.8) digestion at 37°C for 1 h. 80 µg protein loaded onto the stacking gel. **Lane 15:** Pepsin control (75 µg).

**Right panel:** The same gel was subsequently stained with Coomassie Brilliant Blue to confirm protein band patterns observed by TCE fluorescence.

**Supplementary Figure S2: SDS-PAGE characterization of Biova® protein solubility and digestibility.**

Representative 18% SDS–polyacrylamide gel containing TCE for total protein visualization. **L**: molecular weight ladder. **B**: Biova® sample (10 mg/mL in ddH_2_O); 100 µg protein loaded onto the stacking gel. **B + P:** Biova® (10 mg/mL in ddH_2_O) acidified to pH 2 and digested with pepsin at 37°C for 2 h; 100 µg protein loaded. **P:** Pepsin control (19,5 µg). **B + P+1K:** Biova® (10 mg/mL in ddH_2_O) acidified to pH 2 and digested with pepsin at 37°C for 2 h, followed by pancrelipase (1 IU Creon®; pH 8) digestion at 37°C for 2 h. 100 µg protein loaded onto the stacking gel. **1K:** Creon® control (1 IU). **B + P+1K UV:** Biova® (10 mg/mL in ddH_2_O) acidified to pH 2 and digested with pepsin at 37°C for 2 h, followed by pancrelipase (1 IU Creon®; pH 8) digestion at 37°C for 2 h, sterilized by UV light exposure overnight. 100 µg protein loaded onto the stacking gel. **B + P+1K A:** Biova® (10 mg/mL in ddH_2_O) acidified to pH 2 and digested with pepsin at 37°C for 2 h, followed by pancrelipase (1 IU Creon®; pH 8) digestion at 37°C for 2 h, sterilized by autoclave (121°C for 15 minutes), 100 µg protein loaded onto the stacking gel. **B + P+1K F:** Biova® (10 mg/mL in ddH_2_O) acidified to pH 2 and digested with pepsin at 37°C for 2 h, followed by pancrelipase (1 IU Creon®; pH 8) digestion at 37°C for 2 h, sterilized by filtration with a cellulose acetate membrane filter, 100 µg protein loaded onto the stacking gel.
